# Human impact on the microbiological water quality of the rivers

**DOI:** 10.1099/jmm.0.055749-0

**Published:** 2013-11

**Authors:** Emőke Páll, Mihaela Niculae, Timea Kiss, Carmen Dana Şandru, Marina Spînu

**Affiliations:** University of Agricultural Sciences and Veterinary Medicine, Cluj-Napoca, Manastur Street, 3-5, Cluj, Romania

## Abstract

Microbiological contamination is an important water-quality problem worldwide. Human impact on this category of contamination is significant and several human-related activities, and also the population explosion, have affected and are still affecting dramatically the aquatic environment. Extensive industrialization and agriculture have led to increased pollution and hydromorphological changes in many river basins. The Danube river is one of the most affected by these changes where human involvement is undeniable, and subsequently, the Danube Delta Biosphere Reserve became one of the most vulnerable ecosystems. This review is an attempt to analyse the microbiological contamination and to identify the major role human activities play in altering the water quality of the rivers.

## Introduction

Some of the key rivers in Europe are affected by pollution, alteration in the river basin and hydraulic engineering (Schiemer *et al.*, 1999; Besemer *et al.*, 2005). Free suspended bacteria in the water and bacteria associated with suspended materials are quoted amongst pollutants (Noble *et al.*, 1997).

Pathogenic organisms are normal components of all ecosystems, but microbiological contamination with faecal bacteria subsequent to anthropogenic activity is considered to be a crucial issue throughout the rivers and especially in the Danube basin (Bayoumi Hamuda & Patko, 2012). Assessment of surface-water and groundwater quality continues to be of main public interest in the developed world. There is a strong demand for monitoring water quality (Pekárová *et al.*, 2009), therefore the assessment of the presence of pathogenic bacteria in water represents a major concern for human- and animal-health protection (Fey *et al.*, 2004; Straub & Chandler, 2003; WHO, 2002).

The Danube is one of the most important rivers in Europe and in the world. Its delta-like estuary at the Black Sea, the Danube Delta Biosphere Reserve, a paradise for various bird and fish species, was included in 1991 in the United Nations Educational Scientific and Cultural Organization (UNESCO) World Heritage List.

Human and animal pathogens of enteric origin are considered important contaminants of the environment, with transmission through the soil, agriculture, water and sediment (Bonetta *et al.*, 2011). In order to monitor the water quality of the Danube river, riparian countries currently use different methods for microbiological analysis. Bacteria are ideal markers of microbial pollution of surface waters because of their quick response to environmental changes. Faecal coliforms and intestinal enterococci are good indicators for assessing faecal pollution and the potential presence of pathogenic agents, which are mainly caused by untreated sewage originating from agricultural land and pastures.

**Fig. 1.**
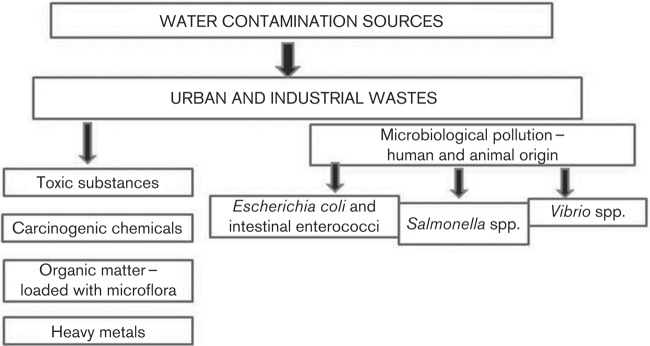
Primary sources of water contamination.

Aquatic ecosystems are currently threatened by human population growth, accompanied by the increased growth of agricultural and industrial activities. Therefore, detection of microbial and genotoxic pollution sources (Fig. 1) is essential for proper watershed management to maintain water traits according to quality goals (Farnleitner *et al.*, 2001; Kolarević *et al.*, 2011).

### General description

Located in central Europe, the Danube basin is ‘the most important non-oceanic body of water’ and flows into the Black Sea through a large delta (Mladenović-Ranisavljević *et al.*, 2012). With a total length of 2857 km from its source at a height of 1078 m in the Black Forest, Germany, to form a delta before the Black Sea in Romania (Helmer & Hespanhol, 1997), the river flows through nine countries and five capitals with 0.5–2 500 000 inhabitants (Winter *et al.*, 2007; Bayoumi Hamuda & Patko, 2012). The watershed of the Danube covers 817 000 km^2^ and drains all or significant parts of Germany, Austria, the Czech Republic, the Slovak Republic, Hungary, Croatia, Slovenia, Bulgaria, Romania, Moldova, Ukraine and parts of the Federal Republics of Yugoslavia, Bosnia and Herzegovina (Helmer & Hespanhol, 1997). Its coastline is almost 240 km long, of which about 75 km represents the coastline of the Kilia distributary delta (Ukraine) and 165 km comprising the Sulina section, the Sfântu Gheorghe distributary delta and the lagoon complex Razim–Sinoie (Romanian territory) (DeltaNet; www.deltanet-project.eu/).

### Sources of pollution

Due to transiting several countries and industrialized urban centres (Kolarević *et al.*, 2011), the waters of the river are exposed to human activities and factories, which contribute to extensive use of the water and therefore pollution. Urban and industrial wastes are often characterized by numerous toxic and carcinogenic chemicals, such as heavy metals, and also organic matter, loaded with microflora, which can contaminate water and enter the food chain, posing considerable danger to public health. A large number of known point-source emissions of municipal, industrial and agro-industrial waste enter the Danube and its tributaries, and are listed in the EMIS emission database 2002 (Kirschner *et al.*, 2009).

The contamination of water resources by faecal pollutants poses significant risks to human and animal health since numerous pathogens are often associated with faeces (Reischer *et al.*, 2008). Thorough investigation of catchment hydrology and pollution dynamics is a prerequisite for successful quantitative microbial evaluation. Long-term monitoring of water quality and seasonal changes in the dynamics of microbial sources is a very important requirement (Reischer *et al.*, 2008). Standard tests based on microbial indicator concentrations are used to protect the environment and prevent consumer exposure to pathogens (Lemarchand & Lebaron 2003; Sugumar *et al.*, 2008; Duris *et al.*, 2009).

Subsequent to the research conducted within ‘The Joint Danube Survey’, the microbiological water quality of the Danube river and its tributaries has been classified on the basis of standard parameters (faecal pollution and organic pollution) in five quality classes: little pollution (class I), moderate pollution (class II), critical pollution (class III), strong pollution (class IV), excessive pollution (class V). Based on samples harvested from Romania (river Danube and Danube delta) strongly faecal (classes III–IV) and moderate to critical organic pollution (classes II–III) were observed. The river Arges is amongst the most contaminated tributaries, which has a significant role in altering the water quality. Similarly, rivers Siret (class III) and Prut (class IV) were significant pollution factors for the Danube (Kavka *et al.*, 2006).

Faecal indicator bacteria such as total coliforms, faecal coliforms (thermotolerant coliforms), *Escherichia coli* and intestinal enterococci (faecal streptococci) are excreted by humans and warm-blooded animals, pass sewage treatment plants in large amounts, and survive, preserving their pathogenicity for a certain time.

### Microbiological pollution

*Salmonella* spp., enteropathogenic *E. coli* and *Vibrio* spp*.*, represent important pathogens. The genus *Salmonella* comprises more than 2400 serotypes, most of which are considered as an endemic public-health concern worldwide (Baker *et al.*, 1983, Baggesen *et al.*, 2000; Soto *et al.*, 2006). *Salmonella enterica* serovar Typhimurium, diagnosed by random amplified polymorphic DNA typing, antimicrobial resistance, and plasmid and integron profiles, represents a common cause of enteric disease in many countries (Soto *et al.*, 2006), and has been involved in water-borne disease of humans and animals (Dondero *et al.*, 1977; Davies, 2001; Kariuki *et al.*, 1999; Mmolawa *et al.*, 2002; Martinez-Urtaza *et al.*, 2004).

Salmonellosis is more commonly associated with contaminated foods and feeds than with waters; nevertheless, salmonellae have frequently been found in effluents from sewage treatment plants, in industrial wastes, and in streams that receive a variety of sewages and industrial wastes (Kampelmacher & Van Noorle Jansen, 1970; Dondero *et al.*, 1977). Frequent isolation of salmonellae from the surface waters of an area gives rise to questions as to the origins of the organisms, their survival or persistence, and their relevance to public health (Kampelmacher & Van Noorle Jansen, 1970). In the Danube river, an increase in the incidence of antibiotic-resistant *Salmonella* strains was identified (Janousková *et al.*, 1975). In order to detect these pathogens it is necessary to assess the virulence factors that have been shown to be relevant to the pathogenesis of *S. enterica* serovar Typhimurium, and endonucleases (*Xba*I and *Bln*I). Detection and mapping of the V genes macrorestiction profiles and their comparison with available data for other strains of *Salmonella* is an established strategy (Soto *et al.*, 2006).

*E. coli* is one of the specific indicators of faecal contamination in tropical and temperate regions. Investigation of the bacterial density of water could provide an approach to assess the reliability of monitoring data (Bayoumi Hamuda & Patkó, 2011). Enterohaemorrhagic *E. coli* have emerged as a serious gastrointestinal pathogen in many countries. Although the mode of transmission is mainly through the consumption of contaminated meat (Mead *et al.*, 1999), outbreaks associated with water-borne enterohaemorrhagic *E. coli* have also been described.

In the Danube river basin total coliforms, faecal coliforms and *E. coli* indicate persistent contamination, with lower values of total coliforms in July and the highest value in August. Variations in these parameters could be spatio-temporarily linked to the number of visitors in this ecosystem (Ajeagah *et al.*, 2012).

*Vibrio cholerae* is a Gram-negative bacterium, native to brackish and estuarine environments, and associated with zooplankton, mainly copepods (Colwell *et al.*, 1977; Huq & Colwell, 1996), and aquatic birds (Ogg *et al.*, 1989). It is a major endemic (Rivera *et al.*, 2003; WHO, 2006) pathogen, responsible for cholera, which mainly affects third world populations, and may cause high morbidity and mortality rates. Ecological studies of cholera and *V. cholerae* have revealed the occurrence of cholera to be correlated with sea surface temperature and sea surface height in endemic areas (Colwell, 1996; Lobitz *et al.*, 2000; Louis *et al.*, 2003). The incidence and severity of epidemics have been linked to salinity, water temperature, turbidity and plankton blooms (Huq *et al.*, 2001; Louis *et al.*, 2003).

*V. cholerae* is a heterogeneous species, with 206 serotypes identified to date, based on thermostable somatic O antigens. Only two serotypes, *V. cholerae* O1 and O139, with two main regions related to pathogenicity – the CTX genetic element and the VC pathogenicity island (VPI) (Karaolis & Kaper, 1999) – have been characterized as toxigenic and identified as the aetiological agent of epidemics. They are less frequently isolated in the aquatic environment than non-O1/non-O139 strains (Colwell *et al.*, 1977; Faruque *et al.*, 1998; Rashed *et al.*, 2012).

In the Danube water, two species dominate: *V. cholerae* and *Vibrio metschnikovii* (Seman *et al.*, 2012). Over 624 strains of *V. cholerae* O1 were isolated in Romania in 1977–1995, the highest number (64 %) originating in the Danube delta area (Israil *et al.*, 1998). In recent years, cholera was positively diagnosed in many patients in Romania, the highest frequency being reported in the Danube delta and its neighbouring districts (Damian *et al.*, 1998; Seman *et al.*, 2012). The major sources of cholera contamination were linked mainly to the drinking of surface water directly from the Danube, followed by delta fish consumption and water consumption from fountains infected with cholera vibrios (Israil *et al.*, 1998; Seman *et al.*, 2012).

Routine isolation from water is not achieved by the conventional culture method, *V. cholerae* is present in natural aquatic environments (Huq & Colwell, 1994) and the role of water in the transmission of *V. cholerae* is well established (Colwell, 1993, 2000; Islam *et al.*, 1994; Chomvarin *et al.*, 2007). Recent molecular biology techniques, including the PCR technique, are used for rapid detection of toxigenic *V. cholerae* in the aquatic environment (Chakraborty *et al.*, 1999; Kapley & Purohit, 2001; Chomvarin *et al.*, 2007). *ctxAB* and *tcpA* genes are known to play key roles in the virulence of *V. cholerae*, especially of serogroups O1 and O139 (Colombo *et al.*, 1997; Faruque *et al.*, 1998; Kovach *et al.*, 1996; Shears, 2001; Singh *et al.*, 2001; Radu *et al.*, 2002; Chomvarin *et al.*, 2007). Fast and precise detection of *V. cholerae* in the drinking water and aquatic environment is imperative for disease management and public-health protection (Huq *et al.*, 1995; Kapley & Purohit, 2001).

Assessment and identification of faecal-origin-specific pathogens is a problem of utmost importance in terms of the safety and protection of drinking-water sources. The isolation of water-borne pathogens is difficult due to methodological limitations. The low bacterial concentration in surface water, high costs and protracted detection technology constitute a major problem (Thompson *et al.*, 2006). PCR, showing a very high specificity and sensitivity (Feder *et al.*, 2001), has become the usual method for the detection of pathogens from various sources, because this method can enhance small amounts of DNA (Touron *et al.*, 2005; Moganedi *et al.*, 2007).

## Conclusion

Many of the world's leading environmental agencies have long centred the focus of their attention on the continued pollution of the Danube river. The main problems that affect the water quality of the river are the high pollution following different human activities and also the population explosion. Today, it is important to improve the water quality of the river ecosystem in order to meet the demand from different sectors and to improve the capacity of the water supply for domestic, agriculture, industry, energy and other uses.

In order to protect this precious natural resource, the Romanian government founded an organization, the Danube Delta Biosphere Reserve, but unfortunately this organization can do little to protect water against pollution and eutrophication. Therefore, multiple measures implemented by all responsible authorities are needed to protect this resource and to avoid damage. The fact that in a remarkable number of sampling sites microbiological parameters alone indicated anthropogenic impacts supports and vindicates the application of microbiological parameters in monitoring programs. Knowledge on microbial pollution in lotic aquatic environments appears essential for decision makers in order to take appropriate measures that result in acceptable river water quality and compliance with national and international quality standards and directives.

In order to improve water quality, it is important to set up a management strategy and plans that provide implementing strategies to address water quality. Similarly, for maintaining overall ecosystem health, it is very important to raise global awareness on the problems associated with faecal pollution of water resources.
